# Cellulosic Ethanol: Securing the Planet Future Energy Needs

**DOI:** 10.3390/ijms9050838

**Published:** 2008-05-17

**Authors:** Clifford Louime, Hannah Uckelmann

**Affiliations:** 1 College of Engineering Sciences, Technology and Agriculture, Florida A&M University, Tallahassee, Florida, USA; E-mail: Clifford.Louime@famu.edu; 2 College of Arts and Sciences, Florida A&M University, Tallahassee, Florida, USA; E-mail: Hannah1.Uckelmann@famu.edu

**Keywords:** Biofuels, Ethanol, Cellulose, Energy Policy

## Abstract

Bioenergy is fairly recognized as not only a necessity, but an inevitable path to secure the planet future energy needs. There is however a global consensus that the overall feasibility of bioenergy will require an integrated approach based on diversified feedstocks and conversion processes. As illustrated in the Brazilian experience, the thrust of any bioenergy program should be centered on the principles and criteria of sustainable production. In general the trends are towards exploiting low value cellulosic materials to obtain high-end value energy products. To this end, it is expected that scientific or technical innovation will come to play a critical role on the future prospects and potential of any bioenergy initiative.

Cellulosic materials are the most abundantly produced organic biopolymers on earth. Each year photosynthetic fixation of CO_2_ yields more than hundred billions tons of dry plant material worldwide and almost half of this material consists of cellulose [1]. Other sources of cellulosic wastes include food crops, grassy plants, residues from agriculture or forestry, and the organic component of municipal and industrial wastes. The value of cellulose as a renewable source of energy and carbon has made cellulose hydrolysis the subject of scientific investigations and industrial interest for many years. Cellulosic wastes may be converted to glucose, soluble sugars, alcohol, single cell protein and other industrially useful chemicals using enzymes such as cellulases. Despite the information available on these enzyme systems and on the structure of plant cell walls, application of this knowledge to cellulose degradation has met with limited success. The limited success may be attributed to at least two factors: 1) the inherent complexity and heterogeneity of native cellulose, and 2) our limited understanding of the basic hydrolysis processes [2]. Therefore, an understanding of the molecular mechanisms underlying cellulose degradation in combination with new and superior enzymes may facilitate increased usage of this valuable renewable resource.

One of the most important features of cellulose as a substrate for microorganisms is its insolubility. Individual cellulose molecules are formed into insoluble cellulose microfibrils through hydrogen binding, thereby creating the very complex and recalcitrant physical structure of cellulose found in plant cell walls, seafood wastes, and industrial processes wastes from the brewery and paper pulp industry [2]. Cellulose molecules are in fact strongly associated through inter- and intramolecular hydrogen bonds and van der Waals forces that result in the formation of microfibrils, which in turn form fibers. These molecules form highly ordered crystalline domains interspersed by more disordered, amorphous regions [2]. The degree of crystal in native cellulose is 60–90%.

**Figure:**
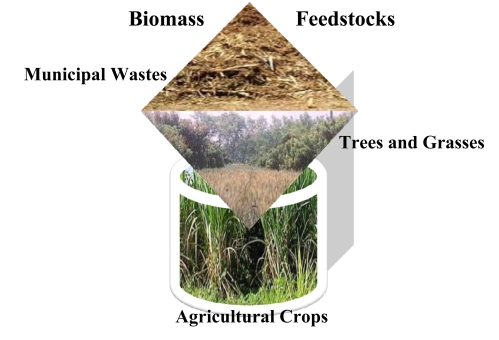


Cellulose almost never occurs alone in nature, but is usually associated with other plant substances. This association affects its natural degradation. All organisms known to convert cellulose efficiently produce a battery of enzymes with different specificities, which act together synergistically. Many workers have recognized that delignification by physical or chemical pre-treatments can greatly enhance the susceptibility of cellulose to enzymatic hydrolysis [3]. Unfortunately, because of their high cost, these pretreatment methods are not feasible at present, especially in developing countries. Furthermore, the processes of both cellulase production and enzyme hydrolysis are also complex. There are many issues still to be resolved both technically (i.e. solid state fermentation) and economically (i.e. substrate prices). For these reasons, direct fermentation processes, which are relatively cost effective, are used for the production of high quality end products that can be obtained by the growth of fungi or bacteria on cellulosic substrates such as wheat or corn straw [4].

The production and use underlying this process have substantial environmental, economic, and security benefits. Biofuels are essentially nontoxic and biodegrade readily. Ethanol can be blended with gasoline (e.g. E85) to increase octane and cut down carbon monoxide and other smog-causing emissions. Every gallon of biofuels used, comparably reduces the hazard of toxic petroleum product spills from oil tankers and pipeline leaks. Using biofuels additionally reduces the risk of groundwater contamination from underground gasoline storage tanks, and runoff of vehicle fuel. The transportation sector is responsible for one-third of the world's carbon dioxide (CO_2_) emissions. Carbon dioxide is the principal greenhouse gas contributing to global warming. Carbon dioxide release from biofuels is largely balanced by CO_2_ uptake for the growth of plants used to produce the fuel [5].

During the last century hydrocarbon feedstocks have dominated industrial inputs. However, reserves of petroleum are finite and, while expected to last well into the 21st century, could be significantly depleted as the world population grows and standards of living improve in developing countries. Utilization of biomass byproducts on a larger scale has the potential to make an impact on reducing reliance on fossil fuels [6]. A strong biomass industry can have tremendous economic benefits including trade deficit reduction, job creation, and strengthening of agricultural markets. Biomass usage can spur the development of new processing, distribution, and service industries in rural communities. Using biomass residues rather than disposing of them in landfills can also reduce a major land use problem. Other economic benefits of a strong biomass industry involve the dependence on petroleum, and the resulting funds transfer to oil-producing countries.

Several health benefits will also come from a cleaner environment. Transportation is the largest single source of air pollution in the world. Harmful pollutants in motor vehicle emissions include carbon monoxide, nitrogen oxides, volatile organic compounds (or hydrocarbons), sulfur oxides, particulates and toxic gases such as benzene. There are a variety of health problems related to exposure to these substances, ranging from eye irritation, to respiratory and cardiovascular illnesses and to cancer [7]. Enormous hidden public health costs come with the use of petroleum for transportation. Reducing the amount of petroleum fuels and replacing them with cleaner-burning biofuels will decrease air pollution and related public health costs.

In term of future prospects of the industry, developments in recombinant DNA technology have enabled researchers to develop cost-effective means of producing selected enzymes and unique enzyme combinations. Highly active fibrolytic enzymes from fastidious microorganisms, such as ruminal fungi, may now be produced in large quantities using biotechnological methods. Plant and animal expression systems are also being developed for the production of recombinant proteins. Genes from microorganisms have not been widely used in these systems to date, but reports of remarkable enzyme activities are generating more interests in this regard [8]. A breakthrough in the investigation of cellulose digestion processes will not only have an enormous impact on the world food supply and economy. It will also greatly influence the types and forms of products produced by the chemical industry and enjoyed by consumers.

## Figures and Tables

**Figure 2.**
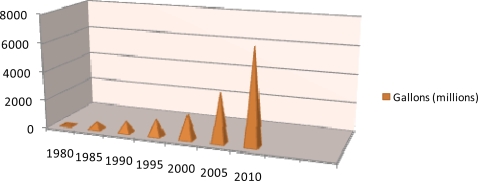
Ethanol Production in the United States.

**Figure 3.**
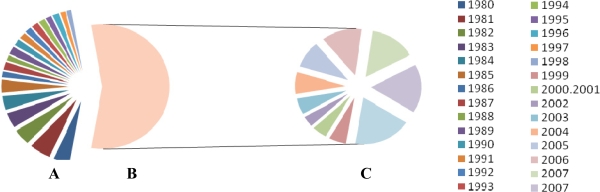
**Direct correlation between crude oil price and ethanol production in the world.** In the past 20 years, hikes in crude oil prices have generated an increase interest in World Production of Ethanol. **A.** Ethanol Production in the years1980–1998 - **B.** Crude Oil Price - **C.** Ethanol production in the years 1999–2007
